# Communication with Relatives and Collusion in Palliative Care: A Cross-Cultural Perspective

**DOI:** 10.4103/0973-1075.53485

**Published:** 2009

**Authors:** Santosh K. Chaturvedi, Carmen G. Loiselle, Prabha S. Chandra

**Affiliations:** 1 Department of Psychiatry, Professor & Head, National Institute of Mental Health and Neurosciences (NIMHANS), Bangalore, India; 2 Department of McGill University Oncology Nursing, Associate Professor, McGill School of Nursing, CIHR PORT Program Leader, Montreal, Quebec, Canada; 3 Department of Psychiatry, NIMHANS, Bangalore, India

**Keywords:** Communication, Collusion, Culture, Palliative care

## Abstract

Handling collusion among patients and family members is one of the biggest challenges that palliative care professionals face across cultures. Communication with patients and relatives can be complex particularly in filial cultures where families play an important role in illness management and treatment decision-making. Collusion comes in different forms and intensity and is often not absolute. Some illness-related issues may be discussed with the patient, whereas others are left unspoken. Particularly in palliative care, the transition from curative to palliative treatment and discussion of death and dying are often topics involving collusion. Communication patterns may also be influenced by age, gender, age, and family role. This paper outlines different types of collusion and how collusion manifests in Indian and Western cultures. In addition, promising avenues for future research are presented.

## INTRODUCTION

Generally speaking, collusion is defined as a secret agreement or cooperation between two or more people who are trying to deceive.[1] In healthcare, collusion implies any information (about the diagnosis, prognosis, and medical details about the person who is ill) being withheld or not shared among individuals involved. Collusion also means that relevant and complete medical information is selectively or not disclosed at all to patients and/or relatives.

Collusion is a universal phenomenon noticed amongst both Western and non-Western societies. Common forms of collusion occur around issues of illness recurrence, deterioration and palliative treatment.[2] Medical teams often collude with patients' relatives to keep the former in the “dark” (e.g., please don't tell him/her about the severity of the illness), or the physicians colluding with patients (e.g., please don't tell my spouse or family about my disease), and not informing the family about the patient's diagnosis or prognosis. Collusion can be manifested through various health professionals (e.g., physicians, nurses, social workers) withholding information from families or the opposite (i.e., families withholding information from healthcare providers due to fear of stigma or discrimination). There are common factors and mechanisms underlying collusion. Nevertheless, ways of handling collusion remain similar across processes and situations.[3]

In India, nearly one half of patients seeking cancer treatment are unaware of their diagnosis or treatment.[4,5] When coming to the medical encounter, most patients are accompanied by a close relative, which often involves a high prevalence of various forms of collusion.

## THE MANY LAYERS AND FACES OF COLLUSION

### Types of collusion

Types of collusion may differ according to broad (e.g., culture) or more focused (e.g., family dynamics) features. Noteworthy is that collusion may be partial or complete. In the early phases of a cancer diagnosis, the family may ‘allow’ the physician to tell the patient about the recommended treatment but ask that the diagnosis not be revealed; or the physician may disclose the diagnosis to the patient but withhold information about prognosis. In palliative care and end-stage disease, the situation can become even more complex. One significant trigger to collusion lies in the transition from curative to palliative treatment. The second issue contributing to collusion is when the topic of death and dying enters into the channels of communication.

“*Collusion is not always a conspiracy of silence, it may be a conspiracy of lopsided communication as well!*”

Authors have written about the ‘recovery plot’ that patients and relatives may follow to spare each other distress and anguish. In addition, they often encourage healthcare providers to rely on this approach as well.[6] The ‘recovery plot’ involves focusing mainly on treatment and recovery issues and not addressing prognosis, long-term disability, distressing symptoms and possibility of relapse, recurrence, and death. The recovery plot may involve the relatives but often physicians focus mainly on treatment issues—what is referred to as ‘medical activism’. This phenomenon of communication is quite common in India, where family members are involved in making the patient *get better* rather than making him or her *feel better*. In the thick of this plot, treating doctors may find themselves being willing allies or they may feel helpless in deconstructing this conspiracy of ‘excessive treatment noise’ and the ‘silence of prognosis and long-term issues’. The recovery plot may be useful for relatives as it gives them pragmatic issues to focus on (e.g., taking the patient for treatment, doing instrumental tasks and providing support). These enabling behaviors are less stressful than dealing with their own and the patient's emotions and confronting the distressing issues related to the patient's illness.

In a qualitative study among patients with small cell lung cancer, researchers in Netherlands found that patients also contribute to the collusion by focusing exclusively on recovery and the ‘treatment calendar’ in their communication patterns with doctors. Even in situations where treatment was in palliative care, relatives and patients worked hard to create a ‘curative aura’ making difficult the physician's wish for straightforward communication. Although all parties individually had occasional doubts about the validity of this plot, they would not verbally acknowledge it, to ensure that they did not undermine others’ trust in future recovery.[6] Such “public” adherence to a recovery plot, however, often cannot be maintained throughout the illness trajectory. When patients relapsed or saw their health status deteriorate, doubts would start being discussed. But even then, patients and relatives would do their best to adhere to the recovery story to spare each other anguish.

## A BITTER PILL OR A SUGAR-COATED ONE? ADVANTAGES AND COSTS OF COLLUSION IN PALLIATIVE CARE SETTINGS

When is open and honest communication harmful? The answer is that it is most often not harmful and may often be beneficial to both patient and relatives. However, some short-term effects of honest communication of prognosis, especially in palliative care settings include emotional outbursts, despair, demoralization and depression. Although these are expected reactions, they are also often accompanied by discussion of unfinished business, emotions and future plans which may ultimately be more fulfilling.

## REGRET AMONG FAMILY MEMBERS ABOUT LACK OF OPEN COMMUNICATION

Although ethnographic studies on the topic of perceptions of the consequences of restricted communication are few in non-Western cultures, studies conducted within the Western world consistently describe regret among family members about not having been open with the patient.[6] Recovery stories and optimism sustained by relatives appear helpful in the initial phases of the illness trajectory, but are found to be painful when it becomes clear that this optimism was based on illusions (i.e., false and unrealistic hope as opposed to realistic hope). Moreover, this state makes it difficult to deal with imminent death as it obstructs “saying farewell” in time and making necessary arrangements. Obviously, this false optimism also hinders patients and relatives in making sensible and well-considered treatment decisions that are not based on avoidance or fear.

However, there may be situations when relatives make the decision to not communicate the exact prognosis to a patient based on past experiences, such as earlier encounters with death (single or multiple) and unresolved related grief. In addition, families may have trouble discussing death due to family conflictual situations or psychological vulnerabilities of family members. Healthcare professionals will assess these situations to gain a comprehensive understanding of family characteristics and dynamics to make an informed judgment about communication patterns to prioritize.

## DO SOCIODEMOGRAPHIC AND OTHER BACKGROUND CHARACTERISTICS MAKE A DIFFERENCE?

Both patients’ and relatives’ methods of communication about prognosis, end of life issues, and death may be determined by several factors which include – patients’ role within the family, age, gender, the family's ability to talk about illness, disability and death in addition to individual emotional vulnerabilities of family members. In fact, studies have documented how these characteristics can modulate communication patterns and illness-related disclosure.

For instance, in a study of advanced colorectal cancer patients aged 70 years or older, one half of the patients did not want information about expected survival and half of them preferred a passive role in treatment decision-making. One-fourth of the patients preferred to leave all decisions regarding treatment to their physicians.[7] However, other than being male, which was associated with a preference for prognostic information, few other clinical or sociodemographic characteristics were significantly associated with patient preferences. Interestingly, the treating oncologists often made errors in judgment when guessing about patients’ preferences for illness-related information. Overall, younger adults with advanced cancer are found to prefer a collaborative role rather than a passive one in decision-making.

Thus, it appears that both patients’ and relatives’ methods of communication about prognosis or end of life issues are determined by several factors which include - patients role in the family, age, gender, the family's ability to talk about illness, disability and death in addition to individual emotional vulnerabilities of family members.

## BREAKING BAD NEWS TO A FAMILY

Various ways and steps of breaking bad news have been formulated primarily within a framework of a one to one interaction between patient and doctor. However, in countries such as India, where filial ties are strong and patients are almost always accompanied by one or more relatives, how should steps of breaking bad news be revised and adapted? Relatives are often reluctant to leave the patient alone with the doctor and they may give strict instructions to the doctor not to reveal the diagnosis. The doctor may be quite comfortable with the ‘recovery plot’ and enters this conspiracy too readily.

It is hence important in filial cultures to guide doctors in breaking bad news with more than one family member present with the patient. More than one relative is involved in the care of the patient and they would all like to know the clinical details. In situations where several family members are present, the health professional may need to identify who the patient thinks is the key relative or the ‘head’ of the family, who can then be involved in the disclosure and discussion process.

## FAMILIAL HIERARCHIES AND ROLES

The role of the elderly in a particular culture may determine the degree of individual versus familial involvement in communication if an older person develops cancer. Some issues in the Indian context are the role of the head of the family (who is often the spokesperson), women being considered to be emotionally weaker and the sons and brothers rather than the daughters, sisters or daughters-in-law being involved in decision-making about their own or their relative's treatment. This is particularly relevant because it is the women who are usually the caregivers and often may not be able to communicate directly with the doctor.

Clayton *et al.*[8] and Parker *et al.*,[9] describe how patients’ and caregivers’ needs may be different at end-of-life. Patients and caregivers were found to be agreeing, however, that the following communication practices were desirable: 1) consistency among different health professionals and openness to questions and discussion, 2) provision of specific information needed to care for the patient, and 3) separate discussions with patient and caregiver.

In developing countries and traditional societies such as India, invariably it is the relatives who ask the doctor and the medical team to provide them (only) with relevant information about the cancer of their relative but not to disclose such information to the patient.[10] Often, the family refuses to let the professionals communicate honestly with the patient. It is commonly alleged that relatives withhold the truth because they cannot face the pain of what is happening and wish to deny it. More commonly, however, it is an act of caring and love for their family member. They cannot bear to upset their loved one who is ill. Family members often try to protect the patient from discussing the nature of the illness which, in turn, hampers effective communication. However, it has been often noticed that patients are quite aware of the nature and severity of the illness and they regularly express their need for open communication.[10]

## WHO ARE WE REALLY PROTECTING? STAFF ISSUES IN COLLUSION

In an experimental study among medical students who were asked to discuss or conceal diagnosis of terminal illness, Panagopoulou[11] found that concealment of diagnosis was much less stressful than disclosing the diagnosis. They hypothesized that doctors often do not disclose diagnosis and go along with the relatives in concealment of important details to protect themselves from their own stress. This stress is most often linked to the handling of emotional reactions following receipt of bad news.

Many nurses and doctors discuss how managing collusion is one of the most difficult issues that they encounter in clinical practice. On the one hand there is the need for telling the truth, while handling the relatives’ reactions to this news often becomes problematic. Sometimes, they too feel that what the relatives are saying may be correct and that disclosure of a realistic prognosis may decrease “healthy” hope.

## EUPHEMISMS AND AVOIDANCE - COLLUSION ABOUT DEATH

If talking about diagnosis and prognosis is difficult for doctors, discussing death is even more challenging. Several studies have shown that doctors hesitate to use the term death or dying and prefer to use euphemisms such as – your time is short; it may be life-threatening, be prepared for the worst, we can only hope now.[12–15]

Whereas these euphemisms are often used to soften the blow, relatives and patients may get confused with issues such as ‘how short will the period of survival be?’ how much hope? how life-threatening?

Although Western cultures have been criticized for viewing death as a failure of medical science rather than as an important natural endpoint in the lifecycle, Eastern cultures are not far behind with the increasing use of technology in medicine and the wish for extending the lifespan through these means. Death has important non-medical meanings and as healthcare professionals, we often seem to overlook such meanings. This avoidance of death and not acknowledging the value of facing this issue to families may lead to restricted communication about this important event. Relatives will seldom initiate discussion about death even if they desire to talk about it. Reasons include the fact that it may be too emotional, they may perceive that the healthcare team does not want to talk about it or that, in some way, talking about death may bring it closer. However, several studies have shown that relatives tend to hold the doctors responsible for this lack of communication following bereavement and feel that honesty would have led to better communication and better outcomes.[13,15] For instance, Lee and Wu[16] discuss how in Singapore, families often collude with the doctor about not discussing death or prognosis with the patient and the negative impact it has on subsequent psychosocial adjustment. They emphasize that while each family may be different culturally, spiritually and emotionally, communication with patients and relatives should be more than a disclosure of diagnosis, prognosis or the dispensing of factual information. It should involve the communication of respect, support, care, concern and availability. Moreover, for many cultures, verbal communication is not the sole source but also includes universal sign language, facial expressions, gestures and attitudes.

The discussion of impending death with family members helps in preparing them for the event with enhanced preparedness related to less mental and physical health negative outcomes post bereavement. When families of terminally ill patients have an opportunity to speak at length with medical staff about their fears, concerns, and questions, they seem better at coping with their loved one's death. A New England Journal of Medicine study[17] reported that longer, more empathetic end-of-life exchanges through conferences eased stress, anxiety, and depression in family members of individuals who died in intensive care units (ICUs). Conducted in 22 ICUs in France, this study randomized families of 126 patients into two groups; one who received short, standard exchanges through conferences, and the other who engaged in more extended sessions and received a brochure on bereavement. In the longer sessions, the staff focused on listening, acknowledging and valuing feelings, encouraging and responding to questions, and gaining an understanding of the patient as a person. When the researchers contacted a representative in each family 90 days later, they found that those who attended longer end-of-life conferences had significantly lower scores on measures of post-traumatic stress, anxiety, and depression than did family representatives from the control group.

Several studies have shown that among caregivers, mental health parameters including depression and grief are worse if they are not prepared for the death of a relative. Preparedness means being ready for death, however, it is not synonymous with anticipatory grief, death acceptance or prognostication.[18] Preparedness may mean different things to different caregivers. For example, it involves (1) knowing what signs and symptoms to expect during the terminal phase (medical), (2) discussing grief and emotions and maintaining relationships with friends and family (psychosocial), (3) prayer and talking about the meaning of the death (spiritual), and (4) planning funeral arrangements (practical). Among the most important predictors of preparedness is healthcare professional-patient/caregivers communication.

Discussion of death and preparedness is a dynamic process, it should occur in stages and be responsive to individuals’ needs so that families can assimilate the information adequately and at their pace. Interestingly, in a survey assessing the above topic, of 988 terminally ill patients and their families, less than 2% of the terminally ill patients reported that completing the initial survey on death and dying caused them a great deal of stress, 7.1% reported some stress, and 88.7% reported little or no stress.[19] Overall, 16.9% of terminally ill patients found talking about death and dying in the interview very helpful, 29.6% found it somewhat helpful, and 49.6% reported little or no help. Slightly more caregivers than terminally ill patients found completing the survey helpful. A total of 19.1% of caregivers found talking about death and dying in the survey very helpful, 34.3% somewhat helpful, and 44.9% of little or no help.

There is indication that whereas individual situations may show some variation, actively engaging in talking about death does not necessarily lead to more distress.

## DEALING WITH COLLUSION ACROSS CULTURES

Accounts of collusion, its manifestation and prevalence vary according to specific personal and contextual factors such as the personality of actors, social network, healthcare services philosophy, as well as the cultural context.

Culture is characterized by “the set of distinctive spiritual, material, intellectual and emotional features of society or a social group, and that it encompasses, in addition to art and literature, lifestyles, ways of living together, value systems, traditions and beliefs”.[20] Understandably, the manners in which patients, families, and healthcare providers communicate and make health-related decisions in palliative stages of an illness are deeply influenced by their socio-cultural context.[21] Different cultures use different strategies for communicating about illness-related factors depending on the local traditions, customs, family ties and family dynamics. This also holds true for the phenomenon of collusion where one finds a complex interplay of local traditions, customs, culturally-bound family relationships and ties and family dynamics.

Issues of collusion related to palliative care have been described in detail in both Indian[5,22] and in Western settings.[23,24] Of particular relevance to palliative care, is the predominance of collusion as individuals are dealing with the physical, emotional, and spiritual issues felt by both the dying patients and the family.[21] Interestingly, when discussing issues of communication and collusion among healthcare providers, relatives and patients, the notion of cultural orientation may be informative. With societies predominantly focusing on individualistic as opposed to a collectivism approach,[25] new insights are gained into the collusion phenomenon. Individualistic societies predominantly favor individuals that are independent, focus on self-interests and develop certain traits and attitudes with a promotion of advantageous relationships. On the other hand, collective societies support interdependent individuals, promote group interests and prescribe social norms and situational constraints with an overarching goal of social harmony. Understandably, the notion of collusion will take on a very different role whether one ascribes to a more individualistic as opposed to a collectivism approach. Perhaps, some of the cross-cultural differences observed in the manifestation of collusion and its ramifications can be best understood from such dichotomous orientation.

The Canadian healthcare system is strongly influenced by other Western systems such as the United States, United Kingdom, Australia, Northern Europe and international institutions such as the World Health Organization and BC (British Columbia) Cancer Agency.[26] These influences have brought discussions from substantive issues of religious affiliation and cultural factors pertaining to patient-healthcare providers’ communication to issues of public policy and ethical debate. Therefore, the ways in which patterns of collusion are dealt with in Canada are increasingly influenced by the complex interplay of these issues. In North America, the most common reasons for healthcare providers' reliance on collusion lies in their lack of confidence in their own communication skills when sensitive patient issues could be disclosed, the second being the need to sustain hope.[2] In many non-Western countries such as in India, the most common form of collusion is between physicians and relatives who seek to keep patients from being aware of their medical status.

Regardless of culture, however, when collusion is identified among family members, attitudes and problems related to selective communication must be addressed such as patients exaggerating the severity of their condition or not being able to discuss unfinished business with family members. While handling collusion or dealing with it, the first step is to acknowledge the collusion and then explore and validate potential reasons for it. The reasons for collusion often lie in the avoidance of adding distress to the patients’ experience or “hurting” them. Relatives are concerned about their family member's wellbeing and feel that the information may be shattering. They often assume that the patient has no clue about his disease, and if he or she does, it will be unbearable. Moreover, the relatives consider their duty to protect their family member who is ill and feel responsible for his or her physical and psychological wellbeing.

However, it is important to establish the potential emotional cost of collusion. Collusion breeds distrust and mistruth which is bound to affect interpersonal relationships among patients and relatives. It can lead to anger in the patient whenever collusion is detected and create an environment of suspicion and mistrust as patients become unsure of what else may be hidden from them.[3,22,27]

### Working through collusion

Possible reasons for collusion and strains related to it need to be established early during the interaction, so that therapists or counselors can explore with relatives or the patient whether they have any idea of what the health status may be or what may be happening to them.[3,22] It should be reinforced that there is no intention of telling the patient without the relatives’ explicit consent and enter into a contract to this effect. The next task is to establish the patient's level of awareness by asking relevant and direct questions which elicits his view of what may be happening to him through the cues provided by the patient. Invariably, patients often show a significant amount of awareness about their health condition in contrast to what the relatives’ perceptions or expectations may be.

This process helps break the barriers between the patient and relatives while improving their interpersonal relationships. It also helps to rebuild trust. It is important to periodically assess the feelings of both patient and relatives while collusion is being dismantled. The pace of handling collusion should be acceptable and tolerable for all involved. While breaking collusion and making links, it is important for professionals to be available when the patient and relative talk to each other. There may be a lot of questions and clarifications requiring straightforward answers. Breaking collusions is often a painful process for counselors and other health professionals because love and care among relatives and the patient become more evident, particularly in the context of an eminent loss. It is important to break collusion in a timely fashion as patients are more likely to be distressed and become morbidly anxious and depressed if left unchecked. This undue distress can lower the threshold at which patients experience physical symptoms such as nausea and pain and cause problems with symptom relief. Failure to deal with important practical and emotional unattended issues also makes it difficult for relatives to work toward resolving their own grief.

## RESEARCH ON COMMUNICATION WITH RELATIVES AND COLLUSION IN PALLIATIVE CARE IN INDIA

More than two decades ago, an interesting study conducted in North India by Gautam and Nijhawan[28] found that although the majority of relatives did not wish the diagnosis to be disclosed to their ill family member, most wanted it to be discussed with them. On the other hand, a majority of the patients preferred to be told of their diagnosis. Literacy level did not significantly affect their responses or opinions. Those patients who did not want to be told of their diagnosis felt that it would make them more depressed or anxious. Relatives who wanted to hide the diagnosis from the patient felt that knowing the diagnosis would make the patient more worried, restless, apprehensive, fearful, and lead to early deterioration of their general condition. The most common reasons provided by those relatives who wanted patients to be told of their diagnosis included a reduction of patients’ fears, apprehension, and curiosity which, in turn, could enhance cooperation in adherence to treatment modalities. In addition, some felt that sooner or later the patients would find out about their disease, so facts should not be hidden from them.

In a study by Muckaden *et al.*,[29] two-thirds of women with cervical cancer had their diagnosis concealed by their husband or family members. The family elders often assumed that the women would be unable to cope with bad news or having to make informed decisions. Interestingly, towards end of life, collusion still persisted only in about 15% of these women.[29] Shubha[30] has discussed this aspect in the Indian and Chinese contexts and asserts that Western individualistic cultures tend to prioritize autonomy and self-determination in end-of-life care, which are reflected in the practices of advance care planning, informed consent, individual decision-making and candid communication of the patient's condition. In contrast, non-Western cultures, such as Indian and Chinese ones, are largely influenced by beneficence and non-maleficence, which promote patients’ welfare and preclude harm to patients.[31] These values cause them to favor patients’ sustenance of hope. Families may want to protect patients by not discussing death and end-of-life decisions directly, whereby encouraging collusion.

## ETHICAL ASPECTS OF COMMUNICATION WITH RELATIVES AND COLLUSION IN PALLIATIVE CARE ACROSS CULTURES

Cancer affects the entire family – not solely the individual diagnosed. Illness-related information is shared among relatives, often irrespective of the person's desire.[22] There are at least two types of dilemmas faced by health professionals regarding communication due to certain cultural constraints - how to break bad news and whom to inform - patient and/or the relatives. The issue of disclosure (or not) is often a significant challenge,[32] due to the unique patient-doctor relationship, with, at times, either the doctor and/or the patient expecting a paternalistic approach. In addition, it is often difficult to decide how much to tell to whom.[33] The few supporters of the “do not tell” policy believe that hope is lost once the truth is out, the “will to live” wanes, and the patient may become depressed. However, when such patients come to know the truth, they may lose trust for not having been told earlier and, it is common to see them manifest feelings of fear, depression, and anger.[34] In due course, cultural constraints observed in non-western societies such as Indian may preclude changes in communication patterns that have now largely taken placed in the western world.[22]

In traditional and developing societies, the family plays a significant role in each stage of healthcare giving - the screening, diagnosis, treatment, and follow-up. In the Indian family scenario, a responsible family member (patriarch) is the decision-maker, who would direct and discuss most treatment-related matters, and invariably, we observe collusion with the healthcare team.[35,36] This paternalistic approach pervades throughout the medical practice and is not confined to end-of-life care. Though this practice stems from the traditional and cultural system of developing societies, it often comes in the way of an individual's autonomy, and deprives him of the benefits of health services and care. On the positive side, collusion may defend the person from potential maleficence, and minimize concerns about the future for the patient. Understandably, relatives often desire to protect their loved ones.[3]

## CONCLUSION

This paper highlights the importance of identifying and dealing with collusion effectively. Clinicians increasingly appreciate the importance of this phenomenon and its potential impact on interaction and communication patterns. Collusion is inevitable in certain cultures and certain contexts but its costs and benefits must be carefully weighed. The imperative to provide culturally competent care (i.e., care that includes a set of behaviors, attitudes, and policies that enables individuals and families from diverse cultural groups to reach their health own health goals) involves a better understanding of issues of collusion and its multiple manifestations across settings and cultures. Research on cultural aspects of collusion, its underlying dynamics, its occurrence, overall impact on wellbeing, psychiatric morbidity, psychosocial adjustment among diverse groups and the relative impact of collusion on the process of providing optimal palliative care must continue to be documented more systematically.
